# Spontaneous Ectopia Lentis in Retinitis Pigmentosa: A Case Report and Review of the Literature

**DOI:** 10.3390/medicina60081281

**Published:** 2024-08-08

**Authors:** Cristina Nicolosi, Giulio Vicini, Lorenzo Beni, Noemi Lombardi, Marco Branchetti, Dario Giattini, Vittoria Murro, Daniela Bacherini, Andrea Sodi, Fabrizio Giansanti

**Affiliations:** 1 Eye Clinic, Neuromuscular and Sense Organs Department, Careggi University Hospital, 50134 Florence, Italy; cristina.nicolosi@unifi.it (C.N.); dr.lorenzobeni@gmail.com (L.B.); noemi.lombardi@unifi.it (N.L.); marco.branchetti89@gmail.com (M.B.); dario.giattini.23@gmail.com (D.G.); vittoria.murro@unifi.it (V.M.); daniela.bacherini@gmail.com (D.B.); andreasodi2@gmail.com (A.S.); fabrizio.giansanti@unifi.it (F.G.); 2 Department of Neurosciences, Psychology, Drug Research and Child Health, University of Florence, 50121 Florence, Italy; 3 Azienda USL Toscana Nordovest, 56121 Pisa, Italy

**Keywords:** retinitis pigmentosa, ectopia lentis, lens dislocation, anterior segment OCT

## Abstract

*Purpose*: We report the successful surgical treatment of a case of spontaneous complete anterior crystalline lens luxation in a patient affected by retinitis pigmentosa (RP), associated with elevated intraocular pressure and pupillary block. Additionally, we review the current literature regarding the association between ectopia lentis and RP. *Case description*: A 44-year-old female RP patient presented to our emergency department reporting severe ocular pain in her left eye (LE) and sickness. She had no history of ocular trauma and did not report systemic disorders. The best corrected visual acuity at presentation was 1/20 in her LE, the intraocular pressure was 60 mmHg, and slit lamp examination showed in her LE a complete dislocation of the lens in the anterior chamber, with mydriasis, atalamia, and a pupillary block. The patient had been administered intravenous mannitol 18% solution and dorzolamide–timolol eye drops and was hospitalized for urgent lens extraction. Anterior segment optical coherence tomography and ultrasound biomicroscopy were performed before surgery. Decompressive 23-gauge pars plana vitrectomy and phacoemulsification were performed, and the capsular bag was removed due to marked zonular weakness, with deferred intraocular lens implant. *Conclusions*: Acute angle closure glaucoma in patients with RP may be rarely caused by spontaneous anterior lens dislocation. To our knowledge, this is the first report of spontaneous anterior lens dislocation in an RP patient, documented through photographs, anterior segment optical coherence tomography, and ultrasound biomicroscopy.

## 1. Introduction

Retinitis pigmentosa (RP) encompasses a broad group of inherited retinal dystrophies, characterized by the progressive degeneration of photoreceptors and the retinal pigment epithelium, which can cause significant visual loss. RP may be associated with different ocular and systemic disorders [1].

RP typically begins with nyctalopia (night blindness) during adolescence, progressing to a concentric narrowing of the visual field, which reflects the primary rod photoreceptors’ dysfunction. Later in life, central vision loss occurs due to the secondary cone photoreceptors’ degeneration. Fundoscopic examination typically reveals mid-peripheral bone spicule-like pigment deposits, waxy pallor of the optic nerve head, and attenuated retinal vessels.

Electroretinography reveals significantly reduced or absent photoreceptor function. Fundus autofluorescence imaging and optical coherence tomography (OCT) demonstrate a progressive loss of outer retinal layers and changes in lipofuscin distribution, displaying characteristic patterns.

Patients with RP frequently experience zonular instability, leading to a higher likelihood of natural crystalline lens or intraocular lens (IOL) dislocation [2]. Consequently, pars plana vitrectomy with IOL implantation is often required to address these cases.

Ectopia lentis (EL), the dislocation of the natural crystalline lens from its normal location, has been rarely reported in association with RP [3]. Lens dislocation is associated with several findings, including reduced vision, monocular diplopia, significant astigmatism, and iridodonesis. Complications of EL may include cataract formation and lens displacement into the anterior chamber or vitreous space. Dislocation of the lens into the anterior chamber or pupil can lead to pupillary block and angle closure glaucoma. Conversely, posterior lens dislocation into the vitreous chamber typically does not cause adverse effects, aside from a significant change in refractive error. The occurrence of angle closure glaucoma secondary to a dislocated lens in RP patients has been described in few reports [4,5,6,7,8,9].

We describe a case of spontaneous complete anterior crystalline lens luxation in a patient affected by RP, associated with elevated intraocular pressure (IOP) and a pupillary block, that was successfully treated by surgical intervention. We also review the current literature regarding the association between EL and RP.

## 2. Case Description

A 44-year-old Italian female RP patient presented to the emergency department of Careggi University Hospital (Florence, Italy) reporting severe ocular pain in her left eye (LE) and sickness. The patient had no history of ocular trauma and did not report systemic diseases. No manifestations of connective tissue disorders, homocystinuria, or other diseases were present. The patient was diagnosed with a classic, nonsyndromic form of RP at the age of 27 years. She had no known family history of RP, although her maternal aunt may have had this retinal dystrophy. Genetic testing has been performed, awaiting its results.

**Figure 1:**
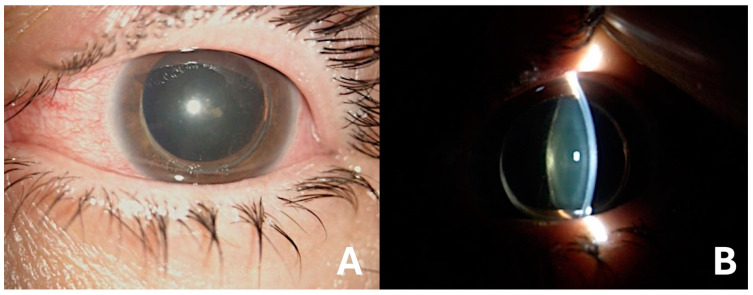
(**A**,**B**) Slit lamp photographs of the patient’s left eye showing the complete dislocation of the lens into the anterior chamber, causing a pupillary block.

The best corrected visual acuity (BCVA) of the patient upon presentation to the emergency department was 1/20 in her LE and 2/10 in her right eye (RE). The IOP measured with Goldmann applanation tonometry was 60 mmHg in her LE and 14 mmHg in her RE. Slit lamp examination showed in her LE a complete dislocation of the lens in the anterior chamber, with mydriasis, atalamia, and a pupillary block (Figure 1). Her RE showed subcapsular cataract, with phacodonesis. Fundus examination of both eyes revealed mild disc pallor, arteriolar attenuation, and bony spicules, indicative of RP.

**Figure 2:**
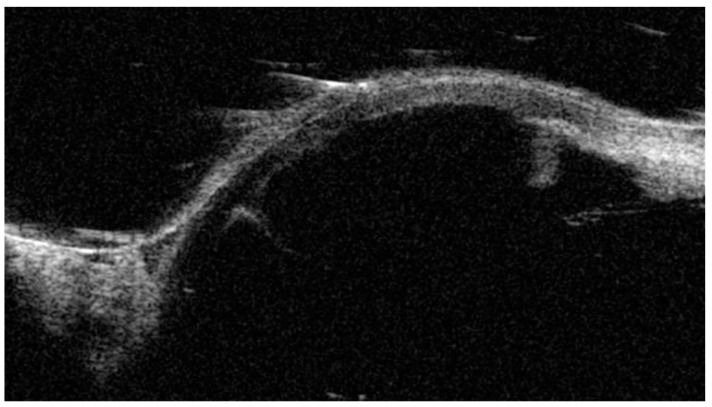
Ultrasound biomicroscopy exam of left eye showing complete crystalline lens dislocation in anterior chamber and normal position of ciliary bodies.

**Figure 3:**
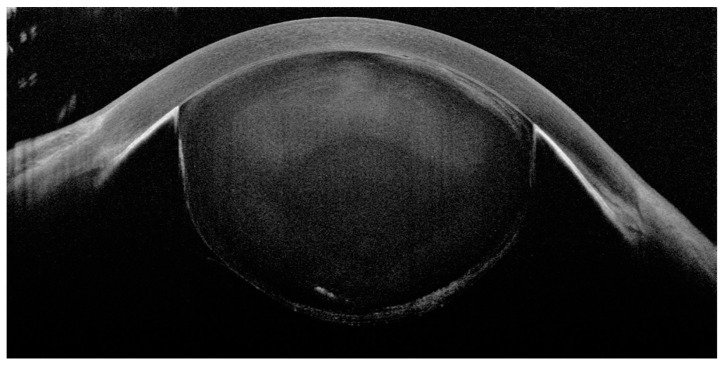
Anterior segment optical coherence tomography (OCT) exam of left eye documented complete crystalline lens dislocation in anterior chamber and corneal–lenticular contact.

The patient had been administered intravenous mannitol 18% solution (500 mL) and dorzolamide–timolol eye drops and was hospitalized for urgent lens extraction. Ultrasound biomicroscopy (UBM) and anterior segment optical coherence tomography (AS-OCT) were performed before the surgery, showing the complete dislocation of the lens in the anterior chamber, anteriorly to the iris diaphragm, and corneal–lenticular contact (Figure 2 and Figure 3). The iris and ciliary body were not visible in the AS-OCT, but appeared normally positioned in the UBM scans, without any rotation or anteriorization. The UBM scan quality was not high because of the difficulty of execution due to the patient’s severe pain and sickness. Axial length measurements were obtained using non-contact partial coherence laser interferometry (IOL Master version 3.01, Carl Zeiss Meditec, Jena, Germany), showing a low axial length in both eyes (19.88 mm in RE and 19.99 mm in LE). The mean keratometric values were 45.5 D and 46.4 D for her RE and LE, respectively. The anterior chamber depth measured in her RE was 2.68 mm. In her RE, the lens thickness was measured to be 3.6 mm (A-scan, Compact Touch, Quantel Medical, version 5.01, Cournon-d’Auvergne, France).

Decompressive 23-gauge pars plana vitrectomy and phacoemulsification were performed, and the capsular bag was removed due to marked zonular weakness. IOL implantation was deferred. The lens had only a mild posterior subcapsular opacity and the phacoemulsification was performed with a low ultrasound rate.

**Figure 4:**
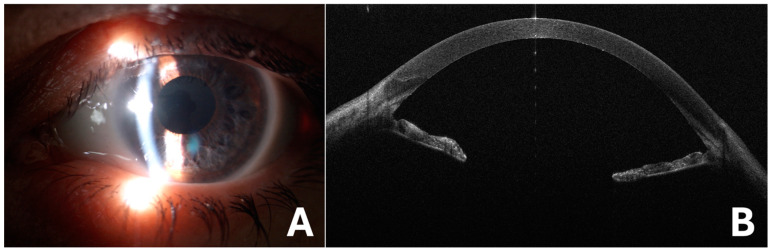
Postoperative anterior segment photograph of left eye (**A**) and anterior segment optical coherence tomography (**B**), showing aphakia and iris atrophy.

The one month postoperative BCVA was 7/10 in her LE, with a correction of +16 sph, and the IOP was 14 mmHg. Postoperative anterior segment photographs and AS-OCT were performed, showing a clear cornea, aphakia, and iris atrophy (Figure 4). A few months later, phacoemulsification was also performed in her RE, without IOL implant, due to phacodonesis. The one month postoperative BCVA was 6/10 in her RE, with a correction of +16 sph, and the IOP was 16 mmHg. The patient is now aphakic and IOL scleral fixation is scheduled in both eyes.

## 3. Discussion

EL is a rare condition associated with a weakening and loss of the zonular fibers that could be found in different ocular disorders, such as traumas, pseudoexfoliation syndrome, heredofamilial diseases including Marfan syndrome, Weill–Marchesani syndrome, Stickler syndrome and Ehler–Danlos syndrome, aniridia, congenital glaucoma, homocystinuria, and RP [3,10]. EL may manifest as an isolated anomaly (simple EL), tipically inherited in an autosomal dominant manner, and may also present with associated pupillary abnormalities in the ocular syndrome known as ectopia lentis et pupillae.

Mutations in the ADAMTSL4 gene (1q21.2), inherited in a recessive manner, and dominant mutations in the FBN1 gene (15q21.1), the same gene that causes Marfan syndrome, have been identified as causes of isolated EL. Mutations in the former are thought to be the most important cause of isolated EL in Europeans. The exact function of these genes has not been clearly established. ADAMTSL4 mutations appear to manifest as a more severe, earlier onset condition than FBN1 mutations.

RP is a condition rarely associated with EL. In RP, the zonular weakness and dehiscence is probably associated to some toxic substances from a long-term inflammatory status and pose a risk of surgery complications [2,11]. The ultrastructure of cataracts in RP is characterized by severe disorganization of the lens fiber that may contribute to the instability of the lens [12].

EL associated with RP has been outlined in a few reports [4,5,6,7,8,9]. Table 1 summarizes the findings of these reports.

The occurrence of angle closure glaucoma secondary to a dislocated cataractous lens in a patient with RP was first described by Helpern et al. in 1981 who reported a bilateral case of EL in a 49-year-old man which required emergency lens extraction [4].

Subsequently, Sato et al. reported two cases of lens dislocation and mild opacities without any intraocular pressure elevation in two siblings with RP and EL with an autosomal recessive inheritance pattern [5]. These two patients were members of a Japanese family with RP, EL, microcephaly, and intellectual disability. There was no history of consanguinity and other systemic disorders in the family.

Yu et al. described a case of bilateral spontaneous EL associated with RP in a patient diagnosed with Marfan syndrome, one of the most common inherited connective tissue disorders [9]. In this case, lens dislocation was not associated with intraocular pressure elevation and the patient underwent phacoemulsification and anterior vitrectomy on both eyes, with deferred scleral fixated IOL implantation 3 months later. EL is considered a major ocular finding in the diagnosis of Marfan syndrome due to the characteristic alteration in connective tissue associated with the disease, and approximately 60% of patients have lens dislocation, while the association of isolated EL with RP is extremely rare and is thought to have a different pathogenesis. In this rare pathological association, it is possible that lens dislocation may also have been favored by the inflammatory changes associated with RP.

The dislocation of the lens may cause angle closure glaucoma due to different types of pupillary blocks: lens subluxation posteriorly to the iris, direct incarceration within the pupil, or complete dislocation into the anterior chamber [13]. The association of acute angle closure glaucoma and anterior lens subluxation in patients with RP has rarely been reported [4,6,7,8].

Wang DD et al. conducted a study investigating the clinical and genetic characteristics of Chinese patients with RP and coexisting angle closure glaucoma [14]. They found that the prevalence of primary angle closure glaucoma (PACG) in RP patients was 2.88%, which is higher than in the general population. This elevated prevalence could be attributed to factors such as nanophthalmos, a thickened lens, EL, or zonular insufficiency. Additionally, patients with a shorter axial length, a greater lens thickness, iridociliary cysts, or nanophthalmos tended to develop PACG earlier. Their genetic analysis identified 30 disease-causing variants across 17 genes in 56.41% of the patients, with PRPH2 being the most commonly mutated gene. These findings underscore the significant association between RP and PACG. The presence of nanophthalmos in patients with RP accounts for most cases of angle closure diseases in RP. In Badeeb et al.’s cohort, RP patients affected by angle closure glaucoma did not exhibit significant hyperopia [15]. Their mean axial length was 23.4 mm, comparable to the normal population, but they had thicker and more anteriorly positioned lenses similar to individuals with PACG.

Ko YC et al. studied the risk of acute angle closure in RP, discovering that patients with RP had an increased risk of acute angle closure compared to controls [16]. Zonular instability is common among patients with RP, potentially leading to anterior displacement of the lens and narrowing of the angle. Additionally, EL and lens subluxation have been identified as factors contributing to angle closure glaucoma in RP patients, particularly after excluding systemic disorders associated with EL. Angle closure glaucoma can occur due to a thickened, anteriorly displaced, or even luxated lens in RP individuals with a normal axial length.

Xu J et al. conducted a study to assess differences in ocular biometric parameters among patients with PACG with and without concurrent RP, aiming to investigate potential associations between the two conditions [17]. A-scan biometry was used to measure lens thickness and axial length. The study included 23 patients with chronic primary angle closure glaucoma (CPACG) associated with RP, 21 patients with acute primary angle closure glaucoma (APACG) associated with RP, 270 patients with CPACG, and 269 patients with APACG without RP for comparison. No significant differences were found in anterior chamber depth, axial length, and relative lens position between patients with PACG associated with RP and those with PACG alone. However, patients with APACG associated with RP exhibited a significantly greater lens thickness compared to those with APACG alone. Patients with PACG associated with RP showed similar ocular biometric characteristics as patients with CPACG and APACG alone. These findings suggest that angle closure glaucoma in RP may not primarily be attributed to these biometric parameters.

Eid TM described a case of RP associated with anterior lens subluxation and acute angle closure glaucoma in a 27-year-old Saudi man developed after pupil dilation [8]. The attack was relieved with maximum antiglaucoma treatment, then two iridotomies were performed, and cataract surgery was scheduled after one month. A Cionni capsular tension ring was used to stabilize and centralize the capsular bag, preventing IOL decentration due to the contracting anterior capsule. Kwon et al. in 2007 reported a case of a 45-year-old male with RP who presented with acute angle closure glaucoma attributed to a relative pupillary block [7]. The crystalline lens was completely dislocated into the anterior chamber, and the patient was surgically treated with anterior vitrectomy, intracapsular cataract extraction, and IOL scleral fixation. Two years later, a similar episode occurred in the patient’s LE, and identical treatment was performed.

Sira et al. reported another case where a 66-year-old Chinese woman, who was slightly myopic and had a history of RP, experienced crystalline lens luxation into the anterior chamber [6]. The patient presented with severe ocular pain and vomiting, with an elevated intraocular pressure of 66 mmHg and visual acuity reduced to no light perception. The lens dislocation caused a pupillary block, necessitating urgent pars plana vitrectomy with lensectomy. The patient was left aphakic. While intraocular pressure normalized postoperatively, visual acuity did not improve. Subsequently, the patient underwent phacoemulsification surgery to remove the cataract in her other eye.

Similarly, in our case, the complete dislocation of the lens in the anterior chamber caused a pupillary block and, consequently, acute angle closure glaucoma.

Acute angle closure glaucoma due to anterior luxation of the lens in RP patients is not a common finding. As far as we know, there are only a few reports of anterior chamber complete lens dislocation, and ours is the first case imaged with both UBM and AS-OCT. These imaging modalities revealed the complete dislocation of the lens into the anterior chamber, positioned anteriorly to the iris diaphragm, with corneal–lenticular contact.

Miura et al. reported the long-term outcomes (three years) of pars plana vitrectomy and IOL implantation in a case series of six eyes of RP patients with lens dislocation, showing that this surgical procedure is safe and has good visual results [18]. The IOL was fixed with suturing in two cases and without suturing in four cases, utilizing flanged intrascleral fixation with the double-needle technique for the sutureless approach. All surgeries were conducted using 25-gauge instruments. Iridectomy was performed in all cases to prevent a reverse pupillary block. The BCVA was maintained or improved at 3 years post-surgery in all six eyes. There were no intraoperative complications reported. The mean deviation of the Humphrey Field Analyzer 10–2 program and the retinal morphology evaluated by OCT showed no abnormal changes before and after surgery. However, in two eyes, the postoperative refractive error was more myopic than the intended refractive correction (greater than 1.5 diopters). The authors concluded that pars plana vitrectomy with IOL implantation can be safely performed in patients with RP, with long-term maintenance of visual acuity. In our case, mannitol solution was administered to reduce the eye pressure, and then, decompressive vitrectomy and phacoemulsification were performed urgently. The LE that showed characteristics of phacodonesis was operated on later in an elective way. In both eyes, the IOL implant was deferred.

## 4. Conclusions

In conclusion, we reported the successful surgical treatment for a case of spontaneous complete anterior crystalline lens luxation in a patient affected by RP associated with elevated intraocular pressure and a pupillary block. As far as we know, this is the first case documented with anterior segment photographs, UBM, and AS-OCT.

Patients with RP are at an increased risk of acute angle closure glaucoma, which may be rarely caused by spontaneous anterior lens dislocation. This condition requires urgent surgical management to remove the dislocated lens. Prompt intervention is critical to prevent further complications and preserve vision.

## Figures and Tables

**Table 1:** Studies reported in the literature regarding ectopia lentis in retinitis pigmentosa patients.

Authors, Year	Sex, Age (Years)	Case Description
Halpern et al., 1981 [4]	M, 49	RP patient presented with bilateral spontaneous dislocation of a cataractous lens into the anterior chamber, resulting in acute pupillary block glaucoma. Both eyes were treated with emergency lens extraction.
Sato et al., 2002 [5]	F, 42	RP patient with microcephaly and intellectual disability. Bilateral ectopia lentis with mild lens opacities was detected. Both lenses were dislocated inferiorly.
	M, 37 (brother of other patient described in the same study)	RP patient with microcephaly and intellectual disability. Bilateral ectopia lentis with mild lens opacities was observed. The lens in the right eye was dislocated upward, while the lens in the left eye was dislocated nasally.
Sira et al., 2005 [6]	F, 66	RP patient presented with acute angle closure glaucoma with a pupillary block by spontaneous anterior lens dislocation. The patient urgently underwent pars plana vitrectomy with lensectomy, resulting in aphakia. Subsequently, cataract surgery was performed in her other eye.
Kwon et al., 2007 [7]	M, 45	RP patient presented with acute angle closure glaucoma with a pupillary block by spontaneous anterior lens dislocation. The patient was surgically treated with anterior vitrectomy, intracapsular cataract extraction, and IOL scleral fixation. Two years later, the same condition occurred in the other eye, and a similar treatment was performed.
Eid et al., 2008 [8]	M, 27	RP patient who developed acute angle closure glaucoma with a pupillary block by anterior lens dislocation after pupil dilation. The attack was relieved with maximum antiglaucoma treatment, then two iridotomies were performed, and cataract surgery was scheduled after one month.
Yu et al., 2013 [9]	M, 32	Patient with RP and Marfan syndrome. Bilateral ectopia lentis with both lenses dislocated superiorly. Intraocular pressure was normal in both eyes. Phacoemulsification and anterior vitrectomy were performed on both eyes. Scleral fixated IOLs were implanted 3 months later.

M: male; F: female; RP: retinitis pigmentosa; IOL: intraocular lens.

## Data Availability

The data presented in this study are available on request from the corresponding author. The data (original imaging) are not publicly available due to privacy issues.
